# Generative AI-based digital storytelling enhances cross-cultural symbol learning through learning engagement

**DOI:** 10.3389/fpsyg.2026.1882907

**Published:** 2026-08-28

**Authors:** Min Yang

**Affiliations:** School of Literature and Communication, Sichuan University of Arts and Science, Dazhou, Sichuan, China

**Keywords:** cross-cultural learning, digital storytelling, educational psychology, generative artificial intelligence, learning engagement, mediation, traditional cultural symbols

## Abstract

**Introduction:**

Cross-cultural learning of traditional cultural symbols is pedagogically demanding because learners must move beyond visual recognition toward historically situated, affectively meaningful, and transferable interpretation. This study examined whether generative artificial intelligence (GenAI)-based digital storytelling improves cultural-symbol learning through learning engagement.

**Methods:**

Study 1 randomized 276 university students to expository text, conventional digital storytelling, or GenAI-based adaptive digital storytelling. Study 2 surveyed 487 students who had recently completed AI-supported cultural learning activities and used confirmatory factor analysis and structural equation modeling to test the proposed engagement pathway.

**Results:**

In Study 1, the GenAI condition produced higher learning engagement, *F*_(2, 273)_ = 24.37, *p* < 0.001, $\eta_{p}^{2}=0.151$, and higher cross-cultural learning outcomes, *F*_(2, 273)_ = 18.92, *p* < 0.001, $\eta_{p}^{2}=0.122$. Engagement partially mediated the GenAI effect (*B* = 0.32, 95% CI [0.19, 0.47]). In Study 2, GenAI storytelling quality predicted engagement (β = 0.61, *p* < 0.001), engagement predicted learning outcomes (β = 0.47, *p* < 0.001), and the indirect effect was significant (β = 0.29, 95% CI [0.22, 0.37]).

**Discussion:**

Across complementary designs, the findings indicate that GenAI-based digital storytelling can support cross-cultural symbol learning by creating more engaging adaptive narrative experiences. Study 2 provides convergent mechanism evidence rather than independent causal proof.

## 1 Introduction

Generative artificial intelligence (GenAI) has become one of the most visible drivers of educational innovation. Recent scholarship increasingly portrays GenAI not only as a tool for content generation, feedback, and tutoring, but also as an environment for adaptive meaning making in which learners interact with multimodal and dialogic representations of knowledge (Kasneci et al., 2023; Yan et al., 2024; Liu and Zhong, 2025). In higher education, this shift is particularly consequential for courses that depend on interpretation, contextualization, and symbolic understanding rather than rote memorization alone (Zawacki-Richter et al., 2019). Traditional cultural learning belongs to this category. When learners encounter culturally dense symbols, motifs, rituals, and artifacts from outside their own lived experience, the challenge is not simply information access but meaningful understanding (Chiu et al., 2023). As a result, the pedagogical question is no longer whether digital tools can deliver cultural content, but whether they can support deeper cross-cultural learning.

Traditional cultural symbols condense historical memory, collective values, aesthetic conventions, and social identity into relatively compact forms. Examples include festival motifs, costume patterns, opera masks, seasonal rituals, calligraphic forms, and material artifacts. For learners from other cultural backgrounds, these symbols often carry a “cultural discount” in the learning process: their surface features are visible, yet their layered meanings are difficult to infer, emotionally distant, or weakly retained. In educational settings, this problem can appear as low interpretive confidence, fragmented comprehension, shallow recall, and poor transfer of cultural knowledge to new contexts. The challenge is therefore psychological as much as curricular. Learners need support in constructing cultural meaning, sustaining attention, and connecting symbolic content to coherent narrative frames (Deardorff, 2006; Shonfeld et al., 2021; Byram, 1997).

Digital storytelling has long been recognized as a pedagogical approach capable of integrating narrative, images, voice, and contextualized sequencing to improve learning and motivation (Robin, 2008; Wu and Chen, 2020). In contrast with decontextualized exposition, storytelling organizes information around causality, perspective, temporality, and human significance. Within educational contexts, such organization can reduce abstraction, increase emotional salience, and foster active interpretation (Mayer, 2009; Green and Brock, 2000). Prior research suggests that digital storytelling is especially suitable for humanities, language learning, and intercultural education because it can render cultural material more memorable and personally meaningful (Wang and Hu, 2025; Szecsi et al., 2025). Yet conventional digital storytelling usually remains fixed once produced. Its narrative pace, examples, explanatory depth, and linguistic scaffolding cannot easily adapt to learners' prior knowledge, language needs, or moment-to-moment misunderstanding.

GenAI changes this pedagogical landscape by enabling adaptive storytelling. A GenAI-based digital story can provide multiple explanation layers, respond to questions, reframe cultural concepts through analogies, generate multilingual support, personalize examples, and dynamically emphasize key symbolic features. From an educational psychology perspective, these affordances matter because they may shape learners' engagement with the task. Learning engagement is a multidimensional construct involving behavioral participation, emotional involvement, and cognitive investment (Fredricks et al., 2004; Bond et al., 2020). It is not a peripheral variable. Rather, engagement is a central mechanism through which instructional design influences learning quality. When learners pay sustained attention, feel interested and connected, and invest effort in interpretation and reflection, they are more likely to comprehend, retain, and transfer knowledge (Bond et al., 2020; Bergdahl et al., 2024). Thus, if GenAI-based storytelling has pedagogical value in cross-cultural learning, one plausible explanation is that it elicits stronger learning engagement than less adaptive formats.

Although the literature on GenAI in education has expanded rapidly, three limitations remain. First, much of the recent discussion focuses on general opportunities, ethical concerns, or students' intentions to use AI tools, rather than psychologically grounded learning outcomes in specific content domains (Kasneci et al., 2023; Zhang and Tur, 2024; Liu and Zhong, 2025). Second, studies on digital storytelling and intercultural learning often demonstrate positive effects, but they rarely isolate the added value of GenAI-enabled adaptivity relative to conventional storytelling (Wu and Chen, 2020; Wang and Hu, 2025). Third, few studies connect GenAI-supported cultural learning to an explicit mediation model rooted in educational psychology. This leaves an important gap: we still know too little about how GenAI-based narrative design translates into improved cross-cultural learning of traditional cultural symbols.

To address this gap, the present article examines the impact of GenAI-based digital storytelling on cross-cultural learning outcomes and tests the mediating role of learning engagement. The research focuses on traditional Chinese cultural symbols because they are pedagogically important yet cognitively demanding for learners who do not share the same cultural background. The study is organized as a two-study investigation. Study 1 uses a randomized experiment to compare expository text, conventional digital storytelling, and GenAI-based digital storytelling. Study 2 employs a survey and structural equation model to validate the proposed mechanism in a broader instructional setting. Three research questions guide the inquiry: (RQ1) Does GenAI-based digital storytelling improve cross-cultural learning outcomes relative to non-adaptive instructional formats? (RQ2) Does GenAI-based digital storytelling enhance learners' behavioral, emotional, and cognitive engagement? (RQ3) Does learning engagement mediate the relationship between GenAI-based digital storytelling and cross-cultural learning outcomes? By answering these questions, the article contributes to educational psychology in three ways. First, it extends research on GenAI from general adoption questions to a psychologically specific learning scenario. Second, it enriches cross-cultural learning research by showing how culturally dense content can be supported through adaptive narrative design. Third, it clarifies learning engagement as a mediating process linking AI-enabled pedagogy to culturally situated learning outcomes.

## 2 Theoretical background

### 2.1 GenAI-based digital storytelling as an instructional form

Digital storytelling refers to the pedagogical use of narrative combined with multimedia elements such as text, image, audio, video, and sequencing to support understanding and expression (Robin, 2008; Wu and Chen, 2020). Educational research consistently shows that storytelling can improve comprehension, motivation, and retention by embedding information in coherent, emotionally meaningful structures (Sadik, 2008; Yang and Wu, 2012). Multimedia learning theory further suggests that learners understand more deeply when words and pictures are organized in ways that guide selection, organization, and integration of information (Mayer, 2009; Moreno and Mayer, 2007). In cultural learning, this principle is especially relevant because learners often need help linking visible features of a cultural symbol to abstract meanings, historical contexts, and social values.

GenAI-based digital storytelling can be understood as an extension of these principles into a more adaptive and dialogic format. Rather than offering one fixed story, a GenAI-supported system can generate explanatory variants, provide contextual analogies, simplify or deepen content, support multilingual access, and maintain narrative continuity while responding to learner questions. Contemporary multimodal large language models are able to process text and visual inputs and generate conversational explanations, which makes them technically suitable for narrative scaffolding in cultural learning tasks (OpenAI, 2024). These affordances align with recent analyses of GenAI in education, which emphasize personalization, scaffolded explanation, and interactive support as major pedagogical opportunities (Kasneci et al., 2023; Yan et al., 2024; Liu and Zhong, 2025). At the same time, the educational value of GenAI cannot be assumed. It depends on whether such adaptivity promotes active and meaningful learning rather than passive consumption. This is why a psychologically grounded mechanism is required.

### 2.2 Cross-cultural learning of traditional cultural symbols

Cross-cultural learning involves more than acquiring factual knowledge about another culture. It includes interpreting symbols, understanding perspective-bound meanings, recognizing culturally situated assumptions, and transferring new knowledge into broader intercultural judgment (Deardorff, 2006; Byram, 1997; Shonfeld et al., 2021). Traditional cultural symbols intensify these demands because they are usually compact carriers of value, memory, and identity. A learner may visually recognize a dragon pattern, lantern motif, opera mask, or festival color scheme without understanding the associated worldview, ritual function, or historical narrative. In other words, symbol recognition does not automatically yield cultural understanding.

This problem can be reframed through the notion of cultural discount in learning. In educational terms, cultural discount appears when materials derived from one cultural system become less legible, less engaging, or less educationally effective for learners from another cultural background. Such attenuation may stem from semantic opacity, contextual distance, limited emotional resonance, or insufficient narrative mediation. Pedagogically, it manifests as low comprehension, low interpretive confidence, and weak transfer. Intercultural education research has long argued that meaningful learning requires learners to encounter culture not as static information but as interpretable, relational, and dialogic content (Deardorff, 2006; Byram, 1997). Digital storytelling provides one route toward this goal, especially when narrative-based activities support intercultural reflection and meaning construction across cultural boundaries (Kahanurak et al., 2023). GenAI may further enhance this route by making symbolic content more adaptive, explorable, and learner-centered.

### 2.3 Learning engagement as a mediating mechanism

Learning engagement is widely conceptualized as a multidimensional construct comprising behavioral, emotional, and cognitive components (Fredricks et al., 2004; Skinner et al., 2009). Behavioral engagement refers to effort, persistence, and participation in learning tasks. Emotional engagement captures interest, enjoyment, and affective connection. Cognitive engagement concerns strategic processing, elaboration, and willingness to invest mental resources in deeper understanding. Across educational contexts, engagement is both an indicator of instructional quality and a proximal driver of learning outcomes (Bond et al., 2020; Bergdahl et al., 2024).

Several reasons suggest that learning engagement should mediate the effect of GenAI-based digital storytelling on cross-cultural learning. First, adaptive storytelling can reduce the psychological distance between learner and material by situating symbols in vivid, coherent, and personally intelligible narratives. Second, interactivity and personalization may increase interest and attentional persistence by supporting learners' perceived autonomy, competence, and meaningful involvement in the learning activity (Deci and Ryan, 2000). Third, the ability to ask follow-up questions and receive tailored explanations can encourage deeper cognitive investment. Recent work on AI-assisted learning also points to the importance of engagement and self-regulated interaction in explaining why some AI-supported experiences improve learning while others merely accelerate superficial completion (Lo et al., 2024; Xu, 2026; Ouyang, 2025). Accordingly, the educational psychology value of GenAI-based storytelling is likely to depend not only on information delivery but also on whether it stimulates meaningful engagement.

## 3 Conceptual model and hypotheses

GenAI-based digital storytelling is proposed to enhance cross-cultural learning outcomes directly by making traditional cultural symbols easier to understand, remember, and apply. It is also proposed to exert an indirect effect through learning engagement. The mediator is conceptualized as a second-order learning process that includes behavioral, emotional, and cognitive engagement, while learning outcomes are defined through symbolic comprehension, contextual interpretation, retention confidence, and transfer-oriented performance. Compared with expository text or non-adaptive digital stories, GenAI-based digital storytelling should provide more responsive and contextually tuned support. Learners can receive narrative clarification, symbolic explanation, multilingual restatement, and example adaptation within the same learning episode. These features should improve the quality of cultural learning. Therefore, we propose:

**H1:** GenAI-based digital storytelling positively predicts cross-cultural learning outcomes regarding traditional cultural symbols.

Instructional formats that are vivid, interactive, and adaptive are more likely to sustain learners' participation, emotional involvement, and cognitive investment than formats that are static and one-directional. Because GenAI can personalize explanations and maintain dialogic continuity within the story, it should elicit stronger engagement. Therefore, we propose:

**H2:** GenAI-based digital storytelling positively predicts learning engagement.

A large body of educational psychology research indicates that engaged learners process information more actively, persist longer, and construct deeper understanding. In cross-cultural learning, these processes should translate into better symbolic interpretation, stronger contextual comprehension, and improved transfer to new scenarios. Therefore, we propose:

**H3:** Learning engagement positively predicts cross-cultural learning outcomes regarding traditional cultural symbols.

If GenAI-based digital storytelling promotes behavioral, emotional, and cognitive engagement, and if engagement in turn enhances learning outcomes, then engagement should transmit part of the instructional effect to the learning result. Therefore, we propose:

**H4:** Learning engagement mediates the relationship between GenAI-based digital storytelling and cross-cultural learning outcomes.

## 4 Materials and methods

### 4.1 Research design and overall logic

The study adopted a two-study design to test both the instructional effect and the underlying psychological mechanism proposed in this article. Study 1 used a randomized between-subject experiment to establish whether GenAI-based digital storytelling produced stronger cross-cultural learning outcomes than two commonly used comparison formats, namely expository text and conventional digital storytelling. This design made it possible to evaluate the incremental value of GenAI-enabled adaptivity rather than the general value of storytelling alone. Study 2 then extended the analysis to a broader instructional setting through survey-based structural equation modeling (SEM), allowing a latent-variable test of the hypothesized mediation path from perceived GenAI-based digital-storytelling quality to cross-cultural learning outcomes through learning engagement.

The two studies served distinct but complementary purposes. Study 1 prioritized internal validity by controlling the learning materials, exposure time, device environment, and task sequence, whereas Study 2 prioritized construct validation and ecological relevance by examining students who had recently completed AI-supported cultural learning activities in actual course contexts. The two samples were collected in different academic terms and from different course sections: Study 1 was conducted during October–November 2024, whereas Study 2 was conducted during March–May 2025. Recruitment rosters were checked before de-identification, and no participant from Study 1 was included in Study 2. Thus, Study 2 constituted a non-overlapping sample rather than a follow-up survey of the experimental participants. The logic of the two-study design was cumulative: if the GenAI-based storytelling condition outperformed the comparison conditions in Study 1, and if the same directional relationship could be reconstructed in a separate sample at the latent level in Study 2, then the argument for a psychologically meaningful instructional mechanism would be strengthened.

### 4.2 Instructional materials and focal learning task

Both studies focused on traditional Chinese cultural symbols that are frequently introduced in intercultural and international education settings but often remain difficult for learners from other cultural backgrounds to interpret. The focal materials covered four symbolic clusters: the dragon motif in festival culture, Peking opera facial masks, traditional knotting patterns, and the Mid-Autumn Festival moon motif. These symbols were selected because they jointly represent visual, ritual, and narrative dimensions of traditional culture, and because they vary in surface familiarity while still requiring contextual explanation for accurate interpretation.

For each symbolic cluster, a common core of information was developed, including historical origin, visual form, social meaning, and an example of contemporary cultural use. The cultural content and focal visual stimulus were held constant across conditions; only the mode of presentation differed. One researcher-curated image was selected for each symbol, and the same four images were used in all three conditions. In the expository-text condition, learners received concise informational passages with the corresponding image embedded on the same screen. In the conventional digital-storytelling condition, the same knowledge core and images were reorganized into a fixed multimedia story with voiceover, sequencing, and image transitions. In the GenAI-based digital-storytelling condition, the corresponding fixed image and narrative section were displayed together within the conversational interface; the image itself was neither generated nor selected dynamically by the model. Only the explanatory dialogue was adaptive, including question-responsive clarification, alternative explanations at different levels of complexity, brief intercultural analogies, optional short multilingual restatement when requested, and context-sensitive elaboration prompts. Participants did not open the images or narrative sections as separate resources. The total instructional time was held within the same 12–15 min exposure window across conditions.

To improve content consistency, all materials were reviewed before deployment against four criteria: conceptual equivalence across conditions, clarity for non-specialist learners, fidelity to widely recognized cultural interpretations, and absence of misleading or overly simplified cultural claims. The GenAI interface then underwent a two-stage pre-deployment validation. First, 24 scripted test sessions (six per symbol cluster) were run using anticipated learner questions at low, medium, and high familiarity levels. Two instructors with experience in cultural communication independently reviewed 96 generated responses using the same accuracy, coherence, boundary-control, and intercultural-responsibility criteria later applied to the study logs. Ninety-three responses (96.9%) met all criteria on the first review; the three flagged responses used analogies that were broader than intended but contained no major factual error. The relevant prompt boundary was tightened and all flagged scenarios passed a second review. Second, 18 graduate students in education and communication completed the full interface as pilot users. Their median completion time was 13.4 min, within the planned exposure window, and no major navigation or display failures were observed. Pilot feedback led to minor changes in wording, pacing, and button labels but did not alter the approved cultural knowledge core or the experimental contrast.

### 4.3 GenAI storytelling implementation and quality control

The GenAI-based storytelling condition was implemented through an API-connected, browser-based experimental interface using GPT-4o as the generative engine (OpenAI, 2024). It did not use the public ChatGPT interface or a Custom GPT. Participants entered the environment through a one-time experimental link opened on the standardized classroom device; they did not need a personal OpenAI account. The system created an isolated anonymous session identifier for each participant and transmitted only the current instructional prompt and learner input required for that session. The interface presented the fixed researcher-approved image, the narrative section, the dialogue box, and the progression controls on the same page. GPT-4o generated text-based clarification and elaboration only; no AI-generated images were used in the experiment.

To ensure that the experimental contrast reflected adaptive narrative support rather than uncontrolled content expansion, the system was constrained by a structured prompt protocol. Each story episode contained: (a) a fixed narrative scene introducing the symbol; (b) a concise explanation of historical and social meaning; (c) an optional learner question; (d) a response calibrated to the learner's self-reported familiarity level; and (e) a brief reflective prompt asking the learner to connect the symbol to an unfamiliar intercultural scenario. The core narrative language was English. When explicitly requested, the system could provide a brief multilingual restatement of a difficult phrase, but the approved cultural content, task instructions, and outcome measures were not changed across languages.

The prompt protocol included explicit rules requiring the model to preserve the approved cultural explanation, avoid unsupported historical claims, distinguish widely accepted interpretations from context-specific examples, and provide short analogies without claiming direct equivalence between cultures. The API temperature was set to 0.30, and each adaptive response was capped at 250 output tokens to limit unnecessary variation and prevent substantially longer exposure than in the comparison conditions. Learners were allowed up to three clarification turns during the activity. To improve reproducibility, the protocol was organized around four fixed prompt blocks: a role-and-boundary block, a symbol-specific knowledge block, a learner-response block, and a reflection block. The role-and-boundary block instructed the system to act as a bounded cultural-learning scaffold rather than an open-ended encyclopedia. The symbol-specific knowledge block contained the approved origin, visual features, meanings, and contemporary use for each cultural symbol. The learner-response block allowed clarification, explanatory-depth adjustment, brief multilingual restatement, and analogy only within the approved knowledge boundary. The reflection block asked learners to explain the symbol to someone from another cultural background. The same prompt blocks and model settings were used for every participant; only learner-provided questions and the resulting bounded clarification varied between sessions.

All generated responses were logged under anonymous session identifiers for quality screening. After data collection, a random 20% sample was independently checked by the same two instructors, neither of whom participated in hypothesis formulation or statistical analysis. The screening focused on cultural accuracy, narrative coherence, alignment with the approved instructional core, boundary control, and absence of unsupported historical generalization. The instructors were not shown participants' learning scores or experimental outcomes during screening. Inter-rater agreement for the retained quality-screening decisions was high (Cohen's κ = 0.88). No response that changed the intended meaning of a symbol or introduced a major cultural error was retained in the analysis. The complete instructional-material summary, deployment configuration, prompt protocol, measurement items, and transfer-scoring rubric are provided in the Appendix.

### 4.4 Study 1: Randomized experiment

#### 4.4.1 Participants

Study 1 included 276 university students recruited during October–November 2024 from course sections in intercultural communication, media literacy, and global education. Participants had diverse disciplinary backgrounds, and all reported that they had not received formal advanced instruction on the specific cultural symbols used in the experiment. The mean age was 20.84 years (*SD* = 1.73); 61.2% were female. The sample included both domestic and international students: 39.5% reported an international or exchange-student background, and 42.4% reported a first language other than Chinese. This composition supported the cross-cultural focus of the learning task while still allowing the experiment to be conducted in a shared instructional setting. Participants were randomly assigned to one of three instructional conditions: expository text (*n* = 92), conventional digital storytelling (*n* = 91), or GenAI-based digital storytelling (*n* = 93). The sample was not treated as a homogeneous group of foreign learners. Rather, the learning task was defined as cross-cultural because all participants were asked to interpret traditional Chinese cultural symbols for culturally diverse audiences and to transfer those interpretations to unfamiliar intercultural scenarios. This design allowed domestic students, international or exchange students, and students with different first-language backgrounds to be evaluated within a common cross-cultural meaning-making task while still permitting subgroup robustness checks based on linguistic and cultural exposure.

An *a priori* power analysis for a one-way ANOVA with three groups indicated that a minimum sample of 207 participants would provide 0.90 power to detect a medium effect size at α = 0.05 (Faul et al., 2007). The final sample exceeded this requirement. Randomization was checked by comparing demographic and baseline variables across the three groups before exposure to the learning materials.

#### 4.4.2 Procedure

The experimental session lasted approximately 30 min and followed the same procedural sequence across conditions. First, participants completed a brief pre-task questionnaire assessing demographic information, prior familiarity with Chinese culture, confidence in understanding English-language explanatory material, and prior experience using AI-supported learning tools. Second, they were exposed to one of the three instructional formats. Third, participants completed the post-task questionnaire measuring learning engagement and perceived learning outcomes, followed by an applied knowledge quiz and a short transfer-scenario task.

The learning environment was standardized as much as possible. Participants completed the task individually in a controlled classroom-laboratory setting using the same type of digital device and were instructed not to consult external resources. Randomization was performed at the participant level by computerized assignment, and the interface delivered standardized written instructions so that condition delivery did not depend on verbal prompting by the researcher. Two trained graduate research assistants supported session logistics and technical troubleshooting but did not provide instructional explanations. Transfer-task responses were exported under coded participant identifiers, randomized in order, and scored with experimental-condition labels removed. The two transfer raters were not involved in prompt design or statistical analysis. Data-cleaning rules for incomplete records and attention checks were applied before condition labels were used in the substantive analyses. At the end of the session, participants were debriefed about the purpose of the study and the distinction between fixed digital storytelling and GenAI-supported adaptive storytelling.

#### 4.4.3 Measures

*Manipulation check*. To verify the instructional distinction between conditions, participants rated perceived adaptivity using three items assessing the extent to which the material adjusted to their understanding, clarified difficult concepts when needed, and provided helpful explanatory support. Responses were recorded on a 7-point Likert scale.

*Learning engagement*. Consistent with educational psychology research, learning engagement was conceptualized as a multidimensional construct comprising behavioral, emotional, and cognitive engagement (Fredricks et al., 2004; Bond et al., 2020). Nine items were used in total, with three items for each dimension. Behavioral engagement captured attention persistence and task focus; emotional engagement captured interest, enjoyment, and felt involvement; cognitive engagement captured interpretive effort, reflective processing, and willingness to connect ideas. The overall scale showed strong internal consistency (α = 0.91). The nine items are reported in the Appendix so that the engagement construct can be evaluated at the behavioral, emotional, and cognitive levels.

*Cross-cultural learning outcomes*. Cross-cultural learning outcomes were operationalized as a combined indicator of subjective symbolic comprehension, contextual interpretation, retention-related confidence, applied quiz performance, and transfer-scenario performance. Six self-report items were adapted from work on intercultural learning and digitally mediated cross-cultural education (Deardorff, 2006; Shonfeld et al., 2021) and showed good internal consistency in Study 1 (α = 0.89). The applied knowledge quiz contained eight items requiring participants to infer the meaning or use of unfamiliar symbol-related scenarios and showed acceptable internal consistency (KR-20 = 0.78). The transfer-scenario task contained two short vignettes in which participants explained how a cultural symbol could be interpreted for learners from another background. Two trained raters scored the transfer responses using a 0–5 rubric for contextual accuracy, interpretive depth, and avoidance of stereotyping. Inter-rater agreement was satisfactory (ICC = 0.86). The scoring rubric distinguished surface recognition, contextual accuracy, interpretive depth, and avoidance of stereotyping; the operational descriptors are reported in the Appendix.

Before combining the three outcome components, their coherence was evaluated using pairwise Pearson correlations and condition-adjusted partial correlations. Because the three components intentionally represented different measurement modalities rather than interchangeable scale items, inter-component association was treated as the primary evidence of coherence. The inferential composite was then calculated as the unweighted mean of the z-standardized self-report subscale, quiz score, and transfer score, ensuring equal contribution despite their different original scales. For descriptive presentation in tables and figures, the composite was linearly transformed to a 1–7-style display metric to facilitate comparison with the self-report measures; all inferential tests involving the composite were conducted using the standardized composite.

*Control variables*. Prior cultural familiarity, English-language confidence, and prior AI-learning experience were included as controls because they may shape both engagement and interpretive performance in cross-cultural learning settings.

#### 4.4.4 Analytical strategy

Study 1 used one-way analysis of variance (ANOVA) to compare the three instructional conditions, followed by Tukey *post-hoc* tests for pairwise comparisons. Effect sizes were reported using partial eta squared ($\eta_{p}^{2}$) for omnibus effects and Cohen's *d* for key pairwise contrasts (Cohen, 1988). Before the composite outcome was used, zero-order and condition-adjusted correlations among the self-report, quiz, and transfer components were examined to verify that the three indicators shared meaningful variance. To test the mediation mechanism, a focal contrast was created in which the GenAI-based digital-storytelling condition was coded 1 and the two non-GenAI conditions were coded 0. Bias-corrected bootstrapping with 5,000 resamples was then used to estimate the indirect effect through learning engagement while adjusting for the control variables (Hayes, 2022). Additional robustness checks examined whether the results remained consistent when the objective quiz score and transfer-scenario score were analyzed separately. Two further checks were added in response to the cross-cultural scope of the study. First, subgroup models examined whether the GenAI advantage remained among students with a first language other than Chinese, students with an international or exchange background, and students with lower prior cultural familiarity. Second, a parallel mediation model separated behavioral, emotional, and cognitive engagement to identify whether the overall mediation effect was concentrated in one engagement dimension or distributed across the three theoretically specified components.

### 4.5 Study 2: Survey-based validation

#### 4.5.1 Participants

Study 2 was conducted during March–May 2025 with university students who had recently participated in AI-supported cultural learning activities embedded in different course sections such as intercultural communication, digital media literacy, Chinese culture in global context, and international education. Recruitment was conducted after the Study 1 roster had been closed, and roster checking confirmed that none of the Study 1 participants entered the Study 2 sample. A total of 512 responses were collected. After removing incomplete responses, patterned responses, and cases failing the attention-check items, the final sample included 487 valid participants. The mean age was 21.16 years (*SD* = 2.04); 59.8% were female. In this sample, 38.8% reported an international or exchange-student background, and 44.1% reported a first language other than Chinese. The sample therefore represented a culturally mixed university-learning context rather than a single-culture convenience group.

#### 4.5.2 Procedure

Participants completed an online questionnaire after being reminded of a recent instructional unit in which AI-assisted narrative resources had been used to explain traditional cultural symbols. To improve recall accuracy, the questionnaire opened with a brief description of the relevant learning scenario and example screenshots of AI-supported narrative materials. Respondents were instructed to answer with reference to that specific learning experience rather than their general opinion of AI. Participation was voluntary and anonymous. The survey platform did not display individual responses to course instructors during data collection, and responses were exported under anonymous case identifiers before analysis.

#### 4.5.3 Measures

All items used a 7-point Likert scale ranging from 1 (*strongly disagree*) to 7 (*strongly agree*).

*Perceived GenAI-based digital-storytelling quality*. Four items assessed the extent to which the AI-supported story was adaptive, vivid, context-sensitive, and helpful for interpreting cultural symbols. The emphasis was not on generic technology liking but on the perceived pedagogical quality of the storytelling experience.

*Learning engagement*. The same three-dimensional logic used in Study 1 was retained. Nine items assessed behavioral, emotional, and cognitive engagement during the AI-supported cultural learning task.

*Cross-cultural learning outcomes*. Six items assessed perceived gains in symbolic comprehension, contextual understanding, interpretive confidence, retention, and transfer to new intercultural situations.

*Control variables*. Prior cultural familiarity, AI literacy, intrinsic interest in the course topic, and first-language background were included as controls because they may independently influence both the mediator and the outcome.

Table 1 summarizes the focal constructs and illustrative items.

**Table 1:** Focal constructs, definitions, and illustrative sample items in Study 2.

Construct	Definition	Illustrative sample item
Perceived GenAI-based digital-storytelling quality	Learner perception that the AI-supported story was adaptive, vivid, context-sensitive, and pedagogically useful for cultural-symbol interpretation	“The AI-supported story adjusted the explanation of cultural symbols to my understanding needs”
Learning engagement	Behavioral, emotional, and cognitive involvement in the cultural-symbol learning activity	“I actively tried to interpret the deeper meaning of the cultural symbols”
Cross-cultural learning outcomes	Perceived gains in symbolic comprehension, contextual understanding, interpretive confidence, retention, and transfer to new intercultural situations	“After the activity, I could better explain these cultural symbols to someone from another background”

Measures were adapted from established work on engagement, intercultural learning, and AI-supported learning (Fredricks et al., 2004; Bond et al., 2020; Deardorff, 2006; Shonfeld et al., 2021; Kasneci et al., 2023).

#### 4.5.4 Measurement quality and common-method assessment

Confirmatory factor analysis (CFA) was conducted before structural testing. Reliability was evaluated using Cronbach's alpha and composite reliability (CR). Convergent validity was assessed with average variance extracted (AVE), and discriminant validity was examined using both the Fornell–Larcker criterion and the heterotrait–monotrait ratio (HTMT) (Fornell and Larcker, 1981; Henseler et al., 2015). Because Study 2 relied on self-report measures, several procedural remedies were implemented in advance, including scale separation, randomized item order, anonymity assurance, and neutral instructions designed to reduce evaluation apprehension (Podsakoff et al., 2003). Harman's single-factor test, marker-variable analysis, and a common-latent-factor comparison were used as statistical checks. These tests suggested that common-method bias was not severe enough to threaten interpretation.

#### 4.5.5 Analytical strategy

The Study 2 analysis proceeded in three stages. First, the measurement model was assessed to verify reliability and construct validity. Second, bivariate correlations were examined to establish the expected associations among the focal variables. Third, SEM with bootstrapped indirect effects was used to test the hypothesized mediation model while accounting for the control variables. Model fit was evaluated using conventional indices, including χ^2^/df, the comparative fit index (CFI), the Tucker–Lewis index (TLI), the root mean square error of approximation (RMSEA), and the standardized root mean square residual (SRMR) (Kline, 2023). Variance inflation factors (VIFs) were also inspected to assess multicollinearity. To avoid overinterpreting the cross-sectional SEM as independent causal proof, Study 2 was used as convergent mechanism evidence and was supplemented with controlled models, subgroup checks, and a parallel engagement-dimension mediation analysis.

## 5 Results

### 5.1 Study 1 results

#### 5.1.1 Baseline equivalence and manipulation check

Table 2 reports the baseline comparison across the three experimental conditions. No significant differences were found in age, prior cultural familiarity, English-language confidence, prior AI-learning experience, or international/exchange-student background, indicating that randomization produced comparable groups before exposure to the instructional materials.

**Table 2:** Baseline equivalence across instructional conditions in Study 1.

Baseline variable	Expository text	Conventional DST	GenAI DST	Test statistic	*p*-value	Result
Age	20.79 ± 1.69	20.91 ± 1.76	20.82 ± 1.75	*F* = 0.11	0.897	Comparable
Prior cultural familiarity	3.36 ± 1.12	3.42 ± 1.08	3.39 ± 1.10	*F* = 0.07	0.932	Comparable
English-language confidence	4.78 ± 0.96	4.83 ± 0.92	4.80 ± 0.95	*F* = 0.06	0.943	Comparable
Prior AI-learning experience	3.71 ± 1.25	3.77 ± 1.18	3.73 ± 1.22	*F* = 0.05	0.951	Comparable
International/exchange background	38.0%	40.7%	39.8%	χ^2^ = 0.14	0.932	Comparable

Continuous variables are reported as means ± standard deviations. DST, digital storytelling.

The three conditions differed significantly in perceived adaptivity, $F_{\left(2,273\right)}=41.62,p<0.001,\eta_{p}^{2}=0.234$. *Post-hoc* comparisons showed that the GenAI-based digital-storytelling condition (*M* = 5.63, *SD* = 0.92) was rated as more adaptive than both the conventional digital-storytelling condition (*M* = 4.79, *SD* = 0.88, *p* < 0.001) and the expository-text condition (*M* = 4.11, *SD* = 0.97, *p* < 0.001). This confirmed the effectiveness of the manipulation.

#### 5.1.2 Coherence of the composite learning outcome

The three components of the Study 1 learning outcome were positively and meaningfully related before standardization and aggregation. Self-reported cross-cultural learning was correlated with applied quiz performance (*r* = 0.46, *p* < 0.001) and transfer-scenario performance (*r* = 0.49, *p* < 0.001), while quiz and transfer performance showed the strongest association (*r* = 0.54, *p* < 0.001). The mean inter-component correlation was 0.50. To verify that these associations were not produced only by the between-condition manipulation, partial correlations controlling for instructional condition were also calculated; the corresponding associations remained significant at *r* = 0.39, *r* = 0.42, and *r* = 0.47, respectively (all *p* < 0.001). Table 3 summarizes these results. The pattern indicates that the three components captured related aspects of cross-cultural learning while retaining distinct subjective, knowledge-application, and transfer information, supporting their use in an equally weighted standardized composite.

**Table 3:** Inter-component coherence of the Study 1 cross-cultural learning outcome (*N* = 276).

Component	1	2	3
1. Self-reported learning subscale	–		
2. Applied quiz score	0.46^***^	–	
3. Transfer-scenario score	0.49^***^	0.54^***^	–

Entries are Pearson correlations before z-standardization. Condition-adjusted partial correlations were 0.39, 0.42, and 0.47, respectively. ^***^p < 0.001.

#### 5.1.3 Main effects on engagement and learning outcomes

Table 4 presents descriptive statistics and omnibus effect sizes. The ANOVA results showed significant differences across conditions for both learning engagement and cross-cultural learning outcomes. Participants in the GenAI condition reported the highest learning engagement, followed by those in the conventional digital-storytelling condition and then the expository-text condition. The same pattern appeared for the composite learning outcome, the applied quiz score, and the transfer-scenario score.

**Table 4:** Descriptive statistics and omnibus effects across instructional conditions in Study 1.

Variable	Expository text (*n* = 92)	Conventional DST (*n* = 91)	GenAI DST (*n* = 93)	*F*	$\eta_{p}^{2}$
Learning engagement	4.02 ± 0.83	4.61 ± 0.79	5.18 ± 0.76	24.37^***^	0.151
Cross-cultural learning composite	4.18 ± 0.86	4.54 ± 0.83	4.93 ± 0.80	18.92^***^	0.122
Applied quiz score (0–8)	5.21 ± 1.42	5.79 ± 1.36	6.48 ± 1.22	19.87^***^	0.127
Transfer-scenario score (0–10)	6.10 ± 1.47	6.78 ± 1.39	7.41 ± 1.25	18.34^***^	0.118
Behavioral engagement	4.11 ± 0.89	4.58 ± 0.84	5.06 ± 0.81	16.84^***^	0.110
Emotional engagement	3.96 ± 0.92	4.66 ± 0.86	5.23 ± 0.80	28.71^***^	0.174
Cognitive engagement	4.00 ± 0.86	4.59 ± 0.82	5.24 ± 0.77	23.46^***^	0.147

Entries are means ± standard deviations. The learning composite is shown on the descriptive display metric; inferential tests used the z-standardized composite. ^***^p < 0.001.

**Figure 1:**
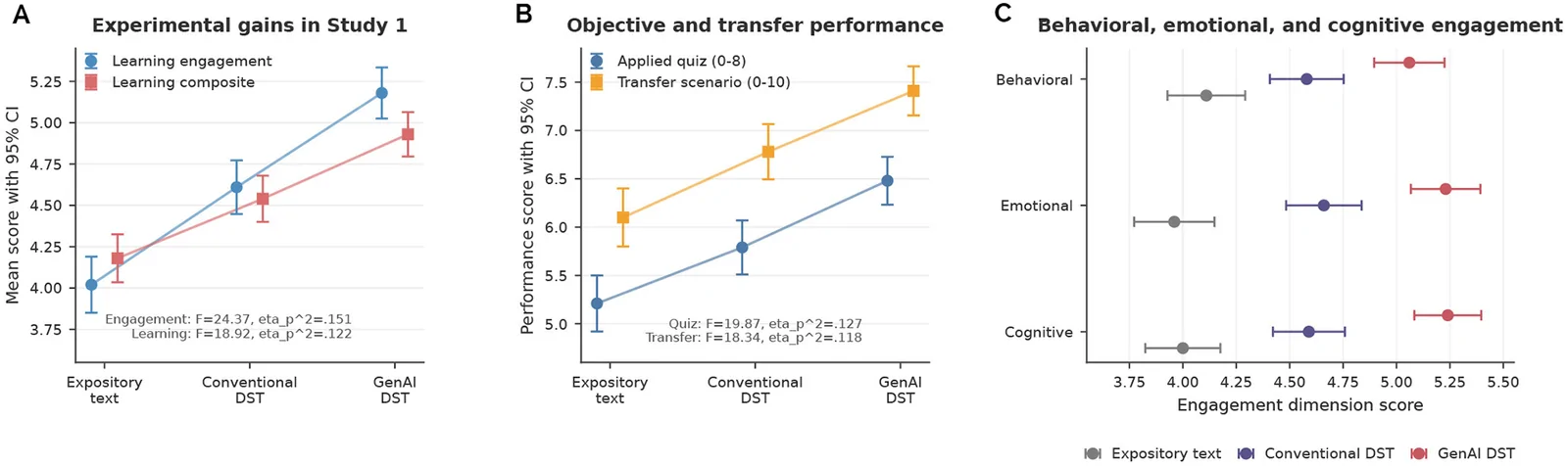
Study 1 experimental effects across instructional conditions. **(A)** Mean learning engagement and cross-cultural learning composite scores with 95% confidence intervals; the composite is shown on the same descriptive display metric as Table 4. **(B)** Applied quiz and transfer-scenario performance with 95% confidence intervals. **(C)** Behavioral, emotional, and cognitive engagement dimension scores across conditions. The visualization shows that GenAI-based digital storytelling produced the strongest gains across both engagement and learning indicators.

Figure 1 complements Table 4 by visualizing the magnitude and consistency of the Study 1 experimental effects. The figure shows a graded pattern across instructional formats: expository text produced the lowest values, conventional digital storytelling produced intermediate gains, and GenAI-based digital storytelling produced the highest engagement and learning scores. Importantly, the same upward pattern appeared not only for self-reported learning engagement and the composite outcome, but also for applied quiz performance and transfer-scenario scoring. The right panel further indicates that the GenAI condition increased all three engagement dimensions, with particularly visible gains in emotional and cognitive engagement. This pattern supports the interpretation that adaptive narrative support improved both learners' subjective involvement and performance-relevant cultural understanding.

*Post-hoc* Tukey tests indicated that the GenAI condition significantly outperformed the text condition on learning engagement (*MD* = 1.16, *p* < 0.001, *d* = 1.46) and the learning composite (display-metric *MD* = 0.75, *p* < 0.001, *d* = 0.90). It also outperformed the conventional digital-storytelling condition on engagement (*MD* = 0.57, *p* < 0.001, *d* = 0.74) and the learning composite (display-metric *MD* = 0.39, *p* = 0.005, *d* = 0.48). The conventional storytelling condition also outperformed the text condition on the learning composite (display-metric *MD* = 0.36, *p* = 0.010, *d* = 0.43) and on engagement (*p* < 0.01). Thus, H1 and H2 were supported in Study 1.

#### 5.1.4 Mediation analysis and robustness checks

Bootstrapped mediation analysis examined whether learning engagement mediated the effect of GenAI-based digital storytelling on cross-cultural learning outcomes. As shown in Table 5, GenAI-based digital storytelling positively predicted learning engagement (*B* = 0.78, *SE* = 0.09, *p* < 0.001). Learning engagement positively predicted cross-cultural learning outcomes (*B* = 0.41, *SE* = 0.05, *p* < 0.001). The direct effect of GenAI-based digital storytelling on learning outcomes remained significant (*B* = 0.27, *SE* = 0.08, *p* = 0.001), and the indirect effect through learning engagement was significant [*B* = 0.32, 95% CI (0.19, 0.47)]. Therefore, H3 and H4 were supported.

**Table 5:** Regression and mediation results for Study 1.

Path	*B*	*SE*	*t*	95% CI	Result
GenAI DST → Learning engagement	0.78	0.09	8.67^***^	0.60, 0.96	Supported
Learning engagement → Learning outcomes	0.41	0.05	8.20^***^	0.31, 0.51	Supported
GenAI DST → Learning outcomes (direct)	0.27	0.08	3.39^**^	0.11, 0.43	Supported
Indirect effect via engagement	0.32	0.07	–	0.19, 0.47	Supported

Control variables included prior cultural familiarity, English-language confidence, and prior AI-learning experience. ^**^p < 0.01, ^***^p < 0.001.

**Figure 2:**
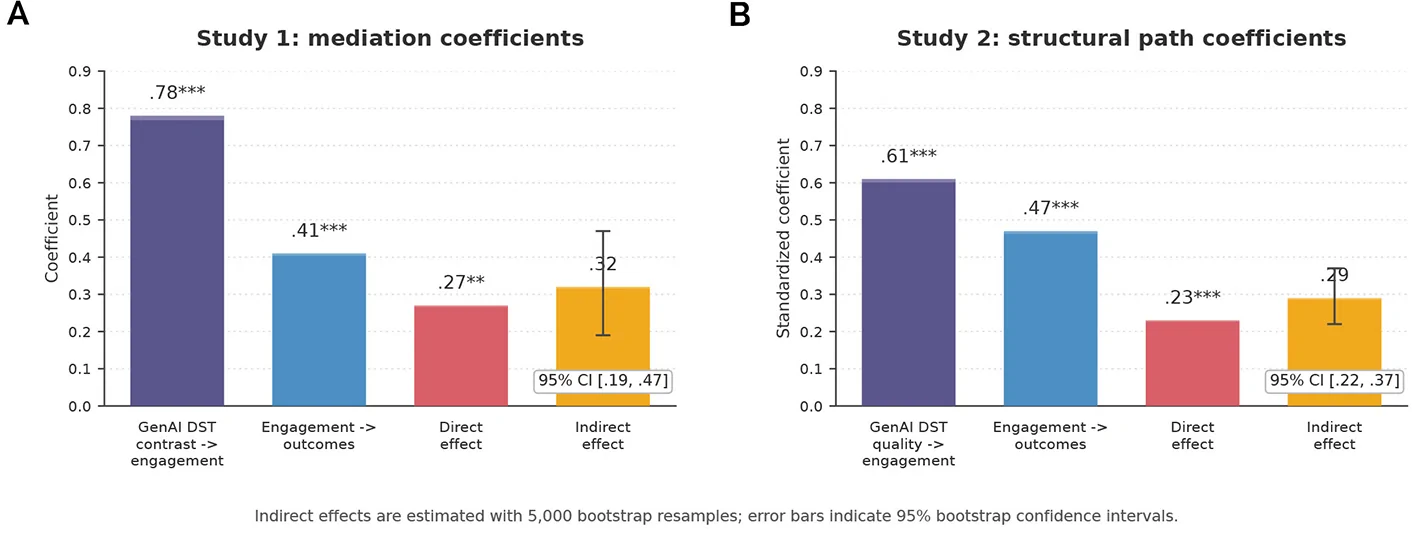
Mediation coefficients across Study 1 and Study 2. **(A)** Study 1 regression-based mediation coefficients for the randomized GenAI digital-storytelling contrast, learning engagement, the remaining direct effect, and the bootstrapped indirect effect. **(B)** Study 2 standardized structural path coefficients for perceived GenAI-based digital-storytelling quality, learning engagement, the remaining direct effect, and the bootstrapped indirect effect. Error bars report 95% bootstrap confidence intervals for the indirect effects. The pattern indicates partial mediation through learning engagement in both studies. **p < 0.01; ***p < 0.001.

Figure 2 summarizes the mediation evidence across the two studies using coefficient-based panels that correspond directly to Tables 5, 6. Panel A visualizes the Study 1 experimental mediation estimates: the GenAI digital-storytelling contrast strongly predicted learning engagement, engagement predicted cross-cultural learning outcomes, and the remaining direct effect indicates partial rather than full mediation. Panel B presents the parallel Study 2 latent-variable estimates, showing the same mechanism at the structural-model level. The side-by-side display makes clear that the engagement pathway was consistently stronger than the remaining direct pathway, supporting learning engagement as the central psychological route through which adaptive storytelling improved cultural-symbol learning.

**Table 6:** Structural path estimates and indirect effect in Study 2.

Hypothesized path	Standardized β	*SE*	*p*-Value	95% CI	*R*^2^ target	Result
GenAI-based DST quality → Learning engagement	0.61	0.05	< 0.001	0.52, 0.70	0.39	Supported
Learning engagement → Learning outcomes	0.47	0.06	< 0.001	0.35, 0.59	0.54	Supported
GenAI-based DST quality → Learning outcomes	0.23	0.05	< 0.001	0.13, 0.33	0.54	Supported
Indirect effect via engagement	0.29	0.04	< 0.001	0.22, 0.37	–	Supported
Prior cultural familiarity → Learning outcomes	0.12	0.04	0.006	0.04, 0.20	–	Supported
AI literacy → Learning engagement	0.10	0.04	0.018	0.02, 0.18	–	Supported

Confidence intervals for indirect effects were estimated with 5,000 bootstrap resamples.

To examine whether the findings depended on the self-report component of the learning composite, two robustness checks were conducted. First, the GenAI contrast remained significant when the applied quiz score was used as the outcome (*B* = 0.52, *SE* = 0.14, *p* < 0.001). Second, the effect remained significant for the transfer-scenario score (*B* = 0.48, *SE* = 0.13, *p* < 0.001). The indirect effects through engagement were also significant for both quiz performance and transfer performance, indicating that the mediation pattern was not driven solely by subjective perceptions of learning.

Because the study concerned cross-cultural learning, additional subgroup checks were conducted to evaluate whether the experimental pattern was limited to one cultural or language group. As shown in Table 7, the GenAI contrast remained positive for engagement and learning outcomes among students with a first language other than Chinese, students with an international or exchange background, students with lower prior cultural familiarity, and domestic students completing the same intercultural transfer task. The estimates were not identical across subgroups, but the direction and significance of the effects were stable, supporting the interpretation that the instructional benefit was not driven by a single sample segment.

**Table 7:** Subgroup robustness of the Study 1 GenAI digital-storytelling contrast.

Subgroup	Engagement effect, *B* (95% CI)	Learning composite effect, *B* (95% CI)	Quiz effect, *B* (95% CI)	Transfer effect, *B* (95% CI)
First language other than Chinese	0.82 (0.58, 1.06)	0.46 (0.27, 0.65)	0.55 (0.24, 0.86)	0.51 (0.22, 0.80)
International or exchange background	0.79 (0.54, 1.04)	0.43 (0.23, 0.63)	0.50 (0.18, 0.82)	0.49 (0.19, 0.79)
Lower prior cultural familiarity	0.86 (0.62, 1.10)	0.48 (0.29, 0.67)	0.57 (0.27, 0.87)	0.53 (0.25, 0.81)
Domestic students in intercultural-transfer task	0.71 (0.50, 0.92)	0.37 (0.20, 0.54)	0.46 (0.20, 0.72)	0.42 (0.17, 0.67)

Estimates are adjusted contrasts comparing the GenAI DST condition with the two non-GenAI conditions. Lower prior cultural familiarity refers to scores at or below the sample median on the pre-task familiarity measure.

### 5.2 Study 2 results

#### 5.2.1 Measurement model

The three-factor measurement model demonstrated good fit: χ^2^/df = 2.31, CFI = 0.956, TLI = 0.949, RMSEA = 0.052, and SRMR = 0.041. As shown in Table 8, all standardized factor loadings exceeded 0.70, CR values ranged from 0.88 to 0.93, and AVE values ranged from 0.59 to 0.72. Discriminant validity was acceptable because each construct's square root of AVE exceeded its correlations with other constructs and all HTMT values were below 0.85.

**Table 8:** Reliability, convergent validity, and discriminant validity statistics in Study 2.

Construct	Loading range	Cronbach's α	CR	AVE	Max HTMT
Perceived GenAI-based DST quality	0.74–0.87	0.89	0.90	0.69	0.68
Learning engagement	0.71–0.86	0.91	0.92	0.59	0.74
Cross-cultural learning outcomes	0.76–0.89	0.90	0.93	0.72	0.76

CR, composite reliability; AVE, average variance extracted; HTMT, heterotrait–monotrait ratio.

Harman's single-factor test indicated that the first unrotated factor explained 32.4% of the variance, below the conventional majority threshold. Adding a common latent factor did not substantially change the standardized path estimates, with changes ranging from 0.01 to 0.04. The marker-variable analysis also produced the same pattern of substantive conclusions. VIF values ranged from 1.18 to 1.67, indicating that multicollinearity was not a concern. Table 9 reports descriptive statistics and correlations.

**Table 9:** Means, standard deviations, and correlations in Study 2 (*N* = 487).

Variable	*M*	*SD*	1	2	3	4
1. Prior cultural familiarity	4.12	1.08	–			
2. Perceived GenAI-based DST quality	5.08	0.93	0.24^***^	–		
3. Learning engagement	5.01	0.88	0.29^***^	0.62^***^	–	
4. Cross-cultural learning outcomes	5.14	0.84	0.31^***^	0.58^***^	0.66^***^	–

^***^p < 0.001.

**Figure 3:**
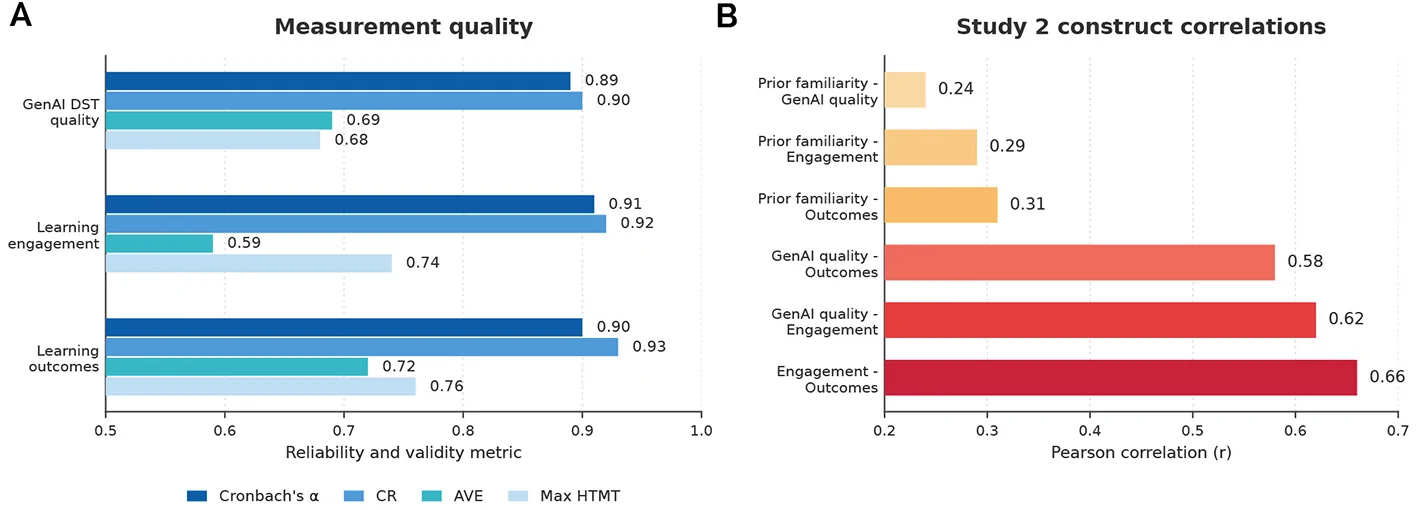
Measurement quality profile and construct-correlation structure in Study 2. **(A)** Profile of Cronbach's alpha, composite reliability, AVE, and maximum HTMT for perceived GenAI-based digital-storytelling quality, learning engagement, and cross-cultural learning outcomes. **(B)** Horizontal correlation-strength visualization of pairwise Pearson correlations among prior cultural familiarity, perceived GenAI-based digital-storytelling quality, learning engagement, and cross-cultural learning outcomes. Bar length and color intensity indicate correlation magnitude. The figure supports adequate measurement quality and shows the expected positive association pattern without implying construct redundancy.

Figure 3 provides a visual summary of the Study 2 measurement evidence and construct associations. Panel A profiles the reliability and validity indices across the three focal constructs, showing that Cronbach's alpha and composite reliability were consistently high, while AVE and maximum HTMT values remained within acceptable ranges for convergent and discriminant validity. Panel B visualizes the pairwise correlation structure as horizontal correlation-strength bars rather than repeating the correlation table. The strongest associations appeared among perceived GenAI-based digital-storytelling quality, learning engagement, and cross-cultural learning outcomes, whereas prior cultural familiarity showed weaker associations. This pattern supports the theoretical model while also showing that the focal constructs were related but not redundant.

#### 5.2.2 Structural model and mediation test

The structural model also fit the data well: χ^2^/df = 2.45, CFI = 0.951, TLI = 0.944, RMSEA = 0.055, and SRMR = 0.044. Perceived GenAI-based digital-storytelling quality was positively associated with learning engagement (β = 0.61, *p* < 0.001), supporting H2. Learning engagement was positively associated with cross-cultural learning outcomes (β = 0.47, *p* < 0.001), supporting H3. The direct path from perceived GenAI-based digital-storytelling quality to cross-cultural learning outcomes remained significant (β = 0.23, *p* < 0.001), supporting H1. Bootstrapping indicated a significant indirect effect through learning engagement (β = 0.29, 95% CI [.22, 0.37]), supporting H4. The model explained 39% of the variance in learning engagement and 54% of the variance in cross-cultural learning outcomes.

A sensitivity analysis was conducted by adding intrinsic interest in the course topic, first-language background, and prior cultural familiarity as additional controls. As shown in Table 10, the mediation pattern remained substantively unchanged. This indicates that the association between GenAI-based storytelling quality, engagement, and learning outcomes was not reducible to general interest in the course or prior cultural exposure.

**Table 10:** Robustness checks for the mediation model in Study 2.

Model specification	GenAI quality → engagement	Engagement → outcomes	Direct effect	Indirect effect
Baseline SEM	0.61^***^	0.47^***^	0.23^***^	0.29 (0.22, 0.37)
With demographic controls	0.60^***^	0.46^***^	0.22^***^	0.28 (0.21, 0.36)
With prior cultural familiarity	0.58^***^	0.45^***^	0.21^***^	0.26 (0.19, 0.34)
With AI literacy and course interest	0.56^***^	0.44^***^	0.20^***^	0.25 (0.18, 0.33)

Values are standardized coefficients. Brackets report 95% bootstrap confidence intervals for indirect effects. ^***^p < 0.001.

**Figure 4:**
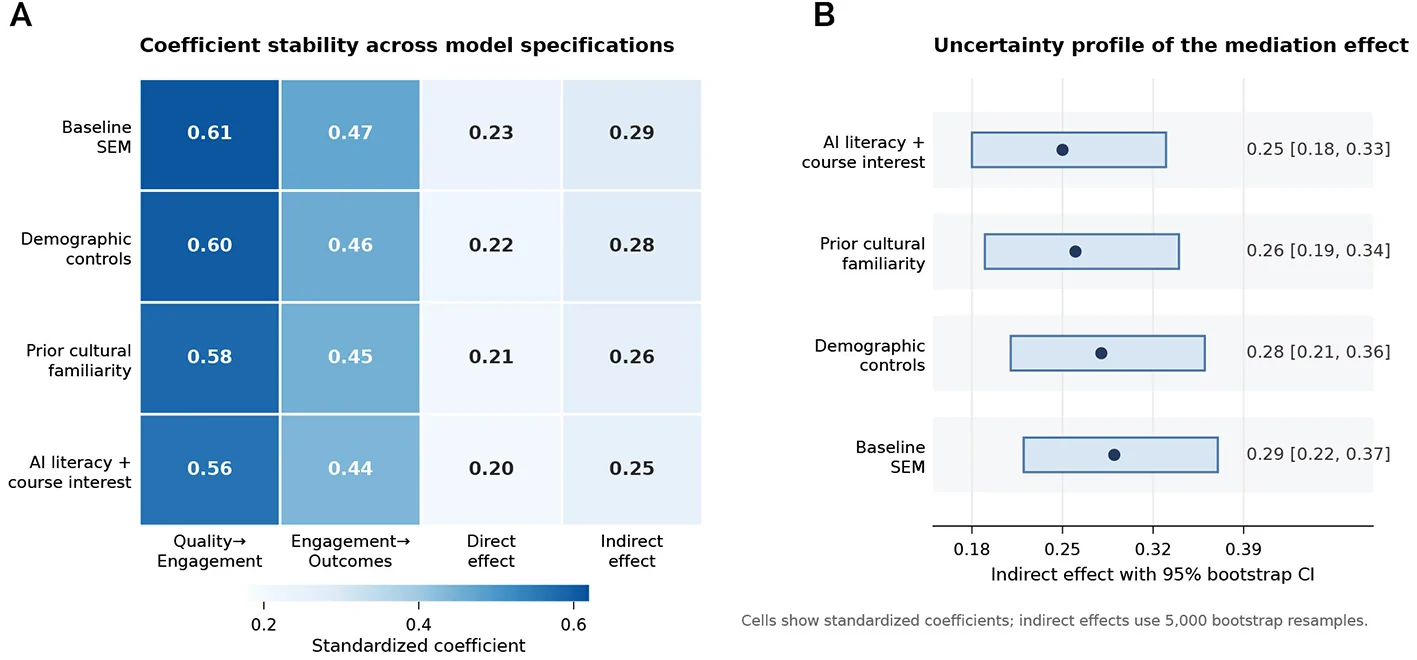
Robustness of the Study 2 mediation model. **(A)** Heatmap of standardized coefficients across the baseline and controlled specifications. **(B)** Indirect-effect estimates with 95% bootstrap confidence intervals. The stable coefficient pattern indicates that the mediation result remained substantively unchanged after adding demographic controls, prior cultural familiarity, AI literacy, and course interest.

Figure 4 visualizes the robustness checks reported in Table 10. The coefficient matrix shows that the quality-to-engagement path remained the largest structural coefficient across all specifications, decreasing only modestly from 0.61 in the baseline model to 0.56 after adding AI literacy and course interest. The engagement-to-outcomes path also remained stable, ranging from 0.47 to 0.44. The indirect effect remained positive across all controlled models, and every 95% bootstrap confidence interval excluded zero. These results indicate that the mediation pathway was not an artifact of demographic composition, prior cultural familiarity, AI literacy, or course interest.

To clarify which component of engagement carried the mechanism, an additional parallel mediation analysis separated behavioral, emotional, and cognitive engagement. As shown in Table 11, all three engagement dimensions contributed to the indirect effect, but the emotional and cognitive pathways were comparatively larger than the behavioral pathway. This pattern is consistent with the theoretical claim that adaptive storytelling supports cultural-symbol learning not only by increasing task focus, but also by strengthening interest, affective involvement, reflective interpretation, and deeper cognitive elaboration.

**Table 11:** Parallel mediation by behavioral, emotional, and cognitive engagement.

Study and mediator	Predictor → mediator	Mediator → outcome	Indirect effect	95% bootstrap CI
Study 1: behavioral engagement	0.52	0.14	0.07	0.03, 0.14
Study 1: emotional engagement	0.81	0.14	0.11	0.05, 0.21
Study 1: cognitive engagement	0.74	0.18	0.13	0.07, 0.24
Study 2: behavioral engagement	0.49	0.15	0.07	0.03, 0.13
Study 2: emotional engagement	0.57	0.18	0.10	0.05, 0.18
Study 2: cognitive engagement	0.55	0.20	0.11	0.06, 0.20

Study 1 coefficients are unstandardized; Study 2 coefficients are standardized. Indirect effects were estimated with 5,000 bootstrap resamples while entering the three engagement dimensions in parallel.

Taken together, the results of Study 2 replicated and strengthened the pattern observed in Study 1 at the latent-variable level. Because Study 2 was survey-based, the findings should be interpreted as evidence consistent with the proposed mechanism rather than as independent causal proof. Nevertheless, the convergence between the randomized experiment and the SEM validation supports the view that learning engagement is a robust explanatory pathway.

## 6 General discussion

The purpose of this research was to determine whether GenAI-based digital storytelling can improve cross-cultural learning of traditional cultural symbols and to explain the mechanism through which such improvement occurs. Across an experiment and a survey-based validation study, the findings converged on a consistent pattern. Compared with less adaptive instructional formats, GenAI-based digital storytelling produced stronger cross-cultural learning outcomes and higher levels of behavioral, emotional, and cognitive engagement. The mediation analyses further indicated that learning engagement served as a substantive explanatory pathway rather than a peripheral accompaniment to good instruction. In other words, the advantage of GenAI-based storytelling was not only that it presented information in a novel way, but that it encouraged learners to stay involved, process cultural content more actively, and connect symbolic material to a more coherent interpretive frame.

These results are noteworthy because traditional cultural symbols represent an especially demanding learning object. Their meanings are rarely exhausted by direct translation or factual description; rather, they depend on historical context, narrative association, ritual function, and culturally shaped perspective. In such circumstances, instructional effectiveness depends not only on whether learners receive information, but on whether they remain sufficiently engaged to interpret layered meaning. The present study suggests that GenAI-based digital storytelling can facilitate this process by turning culturally unfamiliar symbols into adaptive narrative experiences that are easier to follow, more vivid to imagine, and more motivating to explore.

### 6.1 Principal findings

The first important finding is that storytelling alone was beneficial, but GenAI-based storytelling was more beneficial still. In Study 1, conventional digital storytelling performed better than expository text, which is consistent with prior work showing that narrative sequencing and multimodal presentation can improve learning by increasing coherence and salience. However, the GenAI-based condition generated an additional gain beyond fixed storytelling. This pattern indicates that the instructional value of GenAI lies in more than technological novelty. Its added value appears to stem from the capacity to provide responsive explanation, interpretive scaffolding, and adaptive elaboration while preserving a coherent cultural narrative.

The second finding is that learning engagement emerged as a central mechanism across both studies. In the experiment, the GenAI-based condition increased engagement and, in turn, improved cross-cultural learning outcomes. In the survey-based validation, perceived quality of GenAI-based digital storytelling showed a strong positive relationship with learning engagement, which then significantly predicted learning outcomes at the latent-variable level. The additional parallel mediation analysis further suggested that the pathway was distributed across behavioral, emotional, and cognitive engagement, with comparatively stronger emotional and cognitive contributions. This replication across design types strengthens the interpretation that engagement is not incidental. Instead, it is one of the main channels through which culturally adaptive digital narratives become educationally effective.

The third finding is that the effects were not confined to one narrow indicator of learning. The outcome construct covered symbolic comprehension, contextual interpretation, retention-related confidence, applied quiz performance, and transfer-scenario interpretation. This breadth matters because cross-cultural learning cannot be reduced to short-term recall. Learners must also be able to interpret meaning in context and apply that understanding to unfamiliar intercultural situations. The present results therefore suggest that GenAI-based storytelling may support not only information uptake but also a more durable and transferable form of cultural understanding.

### 6.2 Interpretation through educational-psychological theory

The findings can be interpreted productively through three complementary theoretical lenses. First, from the perspective of multimedia learning theory (Mayer, 2009), GenAI-based digital storytelling may reduce unnecessary processing burdens by organizing cultural information into a more coherent representational structure. When learners encounter unfamiliar symbols, they must determine what details are relevant, how those details relate to one another, and why they matter in context. A static or purely expository format places much of that organizational burden on the learner. By contrast, adaptive storytelling can guide attention toward salient features, sequence explanations in a manageable manner, and reframe complex ideas when confusion arises. This does not eliminate cognitive effort, but it can redirect effort from disorganized searching toward meaningful interpretation.

Second, the results align with narrative transportation logic (Green and Brock, 2000). Cultural symbols are more likely to be understood when they are embedded within stories that convey perspective, causality, and social significance. GenAI-based storytelling may deepen this process because it supports dialogue-like elaboration and context-sensitive explanation, thereby sustaining learners' immersion in the symbolic world being presented. Emotional engagement, which rose most sharply in the GenAI condition, is particularly relevant here. When learners feel interested and involved rather than merely informed, symbolic material becomes easier to remember and more meaningful to connect with prior knowledge.

Third, the mediation findings reinforce the central role of engagement in educational psychology (Fredricks et al., 2004; Bond et al., 2020). Engagement is often treated descriptively, as a sign that instruction is going well. The present study supports a more explanatory view. Behavioral, emotional, and cognitive engagement together appear to translate instructional design into learning quality. In culturally complex learning situations, such engagement may be especially important because learners must sustain attention long enough to resolve ambiguity, invest effort in interpretation, and remain affectively open to unfamiliar meaning systems. The contribution of GenAI-based storytelling therefore lies not only in delivering personalized output, but in activating the conditions under which such deep processing becomes more likely.

### 6.3 Theoretical contributions

This study contributes to the literature in several ways. First, it refines research on digital storytelling by distinguishing between fixed digital storytelling and GenAI-based digital storytelling. Earlier work has established that digital storytelling can improve motivation, reflection, and contextual understanding, yet the present research shows that the adaptive capacity introduced by GenAI changes the psychological profile of the learning experience. In this sense, the study moves the field from asking whether stories help learning to asking what kind of stories, under what design conditions, and through which mechanism.

Second, the study extends current GenAI-in-education research beyond adoption attitudes and efficiency-oriented discussions. Much recent work examines whether learners accept AI tools, perceive them as useful, or rely on them in self-regulated learning. Those questions are important, but they do not by themselves explain when GenAI supports meaningful learning. The present article contributes a mechanism-based account by showing that the educational value of GenAI can be theorized and tested at the level of instructional psychology.

Third, the research contributes to cross-cultural and intercultural learning scholarship by offering an educational-psychological explanation for how symbolic distance may be reduced. Traditional cultural symbols often carry what might be described as an interpretive gap: learners can identify visible forms without fully grasping embedded social or historical significance. The results suggest that adaptive narrative design can narrow this gap by increasing interpretive engagement. This offers a useful bridge between intercultural competence research, which emphasizes contextual understanding and perspective taking, and educational design research, which focuses on the architecture of learning experiences.

### 6.4 Implications for educational practice and design

The findings have several practical implications for teaching and instructional design. For course instructors, the study suggests that traditional cultural content should be taught not as isolated facts or decorative examples but as narratively structured, explainable, and discussable symbolic systems. GenAI can support this shift by offering layered explanation, adaptive examples, and responsive clarification when learners encounter difficulty. This may be especially useful in international classrooms where students vary widely in prior cultural familiarity and linguistic confidence.

For curriculum designers, the results imply that the pedagogical question is not whether AI should simply be added to cultural education, but how it should be pedagogically configured. GenAI is most promising when it operates as a bounded interpretive scaffold rather than a substitute for learner effort. Effective design should therefore preserve challenge while reducing confusion. In practice, this means using AI to prompt reflection, compare interpretations, explain symbolic features in context, and support learners in articulating meaning for themselves.

For educational platforms and resource developers, the present findings emphasize the importance of narrative quality. A technically sophisticated AI system may still be educationally weak if its outputs are fragmented, generic, or poorly aligned with learning goals. By contrast, narrative coherence, cultural accuracy, progressive explanation, and interactional responsiveness are likely to be decisive design features. The study thus points toward a more psychology-informed approach to AI-enhanced educational resource development.

### 6.5 Boundary conditions and future directions

The present findings also point to several important boundary conditions. First, the positive effect of GenAI-based storytelling is likely to depend on the quality of instructional orchestration. If the AI-generated story becomes overly long, excessively personalized, or semantically inconsistent, engagement may decline rather than increase. Thus, adaptive capability should not be equated with unlimited variation. In cultural learning, coherence and accuracy remain essential.

Second, learner characteristics may shape the size of the observed effect. Students with higher AI literacy or stronger prior cultural knowledge may use adaptive narrative support more strategically, whereas novices may benefit from stronger guidance but also risk passivity if the system becomes too directive. Future work should therefore examine moderated mediation models involving AI literacy, prior familiarity, cultural openness, language proficiency, or epistemic curiosity.

Third, future research should broaden the range of learning indicators. Although the present work combined self-reported learning outcomes with an applied quiz and transfer task in the experiment, more objective and longer-term indicators would be valuable. Delayed retention tests, transfer tasks scored by external raters, behavioral trace measures, and discourse-based indicators of interpretive depth could all provide a richer account of how GenAI-based storytelling changes learning over time.

Fourth, the current study focused on traditional Chinese cultural symbols and used GPT-4o as the single generative engine. This focus was appropriate for testing the proposed mechanism under a controlled instructional configuration, but the observed effect should not be assumed to be model-invariant or culture-invariant. Language models may differ in instruction following, cultural-knowledge accuracy, multilingual performance, response style, and the stability of bounded dialogue. Future research should therefore replicate the engagement pathway across multiple model families and cultural-content domains, including museum learning, online international exchange, bilingual education, community-based heritage learning, and cultural traditions beyond the Chinese-symbol context. A cross-model-by-domain design would help determine whether the mechanism generalizes even when the surface characteristics of the model and cultural material change.

Fifth, the present GenAI condition should be understood as a bundled adaptive intervention rather than a test of one isolated feature. Dialogic interaction, explanation-depth adjustment, brief multilingual restatement, intercultural analogy, and reflective elaboration were available within the same bounded narrative environment. The manipulation check therefore establishes that learners experienced the condition as more adaptive overall, but it cannot identify which individual feature produced the observed engagement and learning gains. A useful next step would be a factorial component experiment. For example, a 2 × 2 × 2 design could independently vary dialogic responsiveness, adaptive explanation depth, and intercultural analogy while holding the approved cultural knowledge core, images, exposure time, and model constant. Multilingual support could then be tested as an additional factor in a linguistically diverse sample. Such a design would permit estimation of both component-specific effects and interactions among adaptive features.

### 6.6 Limitations

Several limitations should be acknowledged with appropriate caution. First, the samples were drawn from university students, which may limit generalizability to younger learners, adult lifelong learners, or informal education settings. Second, although the subgroup checks supported the cross-cultural interpretation of the task, the study was not designed to compare national or cultural groups as separate populations; larger stratified samples are needed to test whether the mechanism differs by language background, cultural familiarity, and intercultural experience. Third, the survey-based validation relied on self-report data, which can capture psychologically meaningful perceptions but may also reflect shared-response tendencies, and Study 2 was cross-sectional; its SEM results should therefore be interpreted as convergent mechanism evidence rather than causal replication.

Fourth, the GenAI condition bundled several adaptive features within one instructional package. The present experiment shows the incremental effect of the bounded GenAI storytelling configuration relative to fixed formats, but it does not establish whether dialogic interaction, explanation-depth adaptation, multilingual restatement, analogy generation, or reflective elaboration is the principal active ingredient. The overall adaptivity manipulation check cannot resolve component-specific causal contributions, making factorial component experiments an important next step. Fifth, the implementation used GPT-4o and four clusters of traditional Chinese cultural symbols. Differences across language models and cultural domains may alter response accuracy, conversational style, and learner engagement, so the claims should be understood as applying most directly to the tested configuration rather than to all GenAI systems or all cultural-learning content.

Sixth, the study was led by a single author, which concentrates conceptual, design, and analytic responsibility and may leave residual risk of experimenter-related bias. Several safeguards were used to reduce this risk: participant-level assignment was computerized; standardized on-screen instructions reduced researcher discretion during condition delivery; graduate assistants handled session logistics without providing instructional explanations; transfer responses were de-identified, randomized, and scored with condition labels removed; GenAI quality screening was conducted independently by two instructors who did not participate in hypothesis formulation or statistical analysis; and Study 2 responses were collected anonymously. These procedures reduce, but cannot eliminate, researcher-dependence. Future multi-site studies should include independent replication teams and preregistered analysis plans.

Despite these limitations, the convergence of the experimental and SEM findings supports the overall interpretation that GenAI-based digital storytelling can enhance culturally situated learning in a theoretically intelligible way. The results are therefore better understood not as a final verdict on AI-assisted cultural education, but as a strong indication that engagement-centered narrative design deserves a central place in future educational-psychological research on GenAI.

## 7 Conclusion

This study examined whether GenAI-based digital storytelling improves cross-cultural learning of traditional cultural symbols and whether learning engagement explains that effect. Across two complementary studies, the answer was consistently affirmative. Learners exposed to GenAI-based digital storytelling showed stronger behavioral, emotional, and cognitive engagement than those exposed to less adaptive formats, and they also demonstrated better cross-cultural learning outcomes. Learning engagement partially mediated this relationship, indicating that GenAI contributes to learning not only by presenting information in a more accessible form, but by creating the psychological conditions for deeper and more sustained interpretive activity.

The significance of this conclusion extends beyond the immediate context of traditional cultural education. In many current discussions, GenAI is framed primarily as a tool for efficiency, automation, or educational convenience. The present findings point to a more substantive role. When incorporated into a carefully designed narrative environment, GenAI can function as an instructional scaffold that helps learners engage with complex, symbolically dense, and culturally unfamiliar material. This is especially important in cross-cultural learning, where educational success depends on contextual interpretation, perspective taking, and meaningful connection rather than surface-level recognition alone.

For educational psychology, the study offers a mechanism-based explanation linking adaptive narrative design, engagement, and learning quality. For practice, it suggests that the future value of GenAI in education will depend less on its novelty than on whether it is used to support pedagogically coherent, psychologically engaging, and culturally responsible learning experiences. In this sense, GenAI-based digital storytelling is best understood not as a technological shortcut, but as a promising design pathway for fostering richer intercultural understanding in contemporary education.

## Data Availability

The raw data supporting the conclusions of this article will be made available by the authors, without undue reservation.
