# Supplementary material for: Generative AI-based digital storytelling enhances cross-cultural symbol learning through learning engagement
Source: Front Psychol. 2026 Aug 28;17:1882907. doi: 10.3389/fpsyg.2026.1882907 (PMC13562057; doi:10.3389/fpsyg.2026.1882907)
Supplement: Supplementary file 1 [file Data_Sheet_1.pdf]

Supplementary Material

APPENDIX

Appendix A. Instructional Materials and Chinese Cultural-Symbol Application

This appendix documents the instructional materials, adaptive storytelling protocol, measurement items, transfer-scoring rubric, and quality-screening criteria used in the two studies. The purpose of including these materials is to make the learning intervention more transparent and to clarify how the experimental contrast was controlled. The appendix also explains how the materials can be applied to Chinese cultural-symbol instruction in intercultural education settings.

Table A1. Focal cultural-symbol clusters and controlled instructional core.

Symbol cluster	Controlled knowledge elements	Interpretive learning goal	Transfer prompt focus
Dragon motif in festival culture	Visual form, auspicious meaning, collective celebration, historical continuity	Distinguish symbolic auspiciousness from literal animal imagery	Explain why a dragon motif can represent vitality and collective aspiration in a festival setting
Peking opera facial masks	Color conventions, role types, stylized performance, moral and affective cues	Link visual codes to characterization and performance context	Interpret a mask pattern for learners unfamiliar with opera conventions
Traditional knotting patterns	Interlaced form, continuity, blessing, gift-giving and decorative use	Connect material form to social meaning and relational symbolism	Explain how a knot pattern can communicate continuity or goodwill without direct verbal explanation
Mid-Autumn moon motif	Moon imagery, reunion, seasonal ritual, family memory and poetic association	Move from object recognition to ritual and emotional meaning	Explain how the moon motif can express shared memory and reunion across cultural contexts

Interpretive analysis. Table A1 shows that the four symbol clusters were not selected as isolated cultural examples. They were chosen to represent complementary forms of Chinese cultural meaning: festival symbolism, performance coding, material-artifact symbolism, and seasonal ritual memory. Each cluster contained a controlled knowledge core so that the three instructional conditions differed in presentation format rather than in basic cultural content. This structure helped preserve comparability across expository text, conventional digital storytelling, and GenAI-based adaptive storytelling.

Application to Chinese cultural-symbol teaching. In Chinese cultural education, the same structure can be used to design lesson units that move learners from visible form to contextual interpretation. For example, teachers can first present a symbol's visual features, then explain its social or historical meaning, and finally ask learners to translate that meaning for an audience from another cultural background. This approach is especially useful for Chinese culture courses, international communication courses, museum education, and Chinese-as-a-foreign-language activities in which learners need to explain symbols respectfully rather than merely recognize them.

Appendix B. GenAI Storytelling Protocol and Instructional Control

Table A2. Deployment configuration of the GenAI storytelling condition.

Configuration element	Operational specification
OpenAI environment	GPT-4o accessed through an API-connected browser-based experimental interface; neither the public ChatGPT interface nor a Custom GPT was used.
Participant access	One-time experimental link on standardized classroom devices; no personal OpenAI account required; one isolated anonymous session per participant.
Model settings	Temperature = 0.30; maximum 250 output tokens per adaptive response; maximum three learner clarification turns; fixed prompt blocks for all participants.
Narrative delivery	Fixed researcher-approved narrative core with adaptive text clarification, explanation-depth adjustment, brief multilingual restatement on request, intercultural analogy, and reflection prompts.
Image delivery	One fixed researcher-curated image per cultural-symbol cluster, identical across conditions and embedded within the same interface; no AI-generated or dynamically selected images.
Session logging	Anonymous session identifier, prompt/response text, and interaction timestamps retained for quality screening; participant names were not included in the GenAI log.
Pre-deployment validation	24 scripted test sessions reviewed by two independent instructors, followed by full-interface pilot testing with 18 graduate students.
Post-deployment quality control	Random 20% of generated responses independently screened by two instructors; Cohen's $\kappa = .88$.

Interpretive analysis. Tables A2 and A3 clarify both the technical deployment and the pedagogical boundaries of the GenAI condition. The intervention was delivered through a controlled API-connected interface rather than an unrestricted consumer chatbot, while fixed images, fixed knowledge elements, response-length limits, and common prompt blocks were maintained across participants. The protocol required the model to preserve the approved knowledge elements, maintain narrative coherence, provide bounded adaptive clarification, and include transfer-oriented reflection. These controls reduce the risk that the GenAI condition differed from the comparison conditions simply by providing longer, broader, or visually different content.

Application to Chinese cultural-symbol teaching. For Chinese cultural-symbol instruction, this protocol can guide teachers in using GenAI as a narrative assistant rather than as an unrestricted answer generator. A teacher can prepare an approved cultural explanation for each symbol, require the AI system to remain within that explanation, and use adaptive clarification only to adjust language difficulty, analogy choice, and reflective prompts. In practice, this makes GenAI suitable for international classrooms where learners differ in Chinese-language proficiency, prior cultural familiarity, and intercultural experience.

Appendix C. Measurement Items

Interpretive analysis. Table A4 reports the item content underlying the focal constructs. The engagement items distinguish behavioral persistence, emotional involvement, and cognitive elaboration, which is consistent with the theoretical model tested in the article. The perceived GenAI-based storytelling quality items focus on pedagogical quality rather than general technology liking. The learning-outcome items

Table A3. Structured GenAI storytelling protocol used in the adaptive condition.

Protocol block	Function	Operational instruction
Role and boundary	Maintain a bounded instructional role	Provide culturally responsible explanations using only the approved knowledge elements; avoid unsupported historical claims and avoid presenting analogies as direct equivalences between cultures.
Narrative scene	Introduce the symbol through a brief story context	Present the symbol in a short learning scene that includes visual features, social use, and one historically situated meaning.
Adaptive clarification	Respond to learner questions without expanding beyond the approved instructional core	If the learner asks for clarification, restate the meaning at a simpler or more advanced level and connect it to the learner's stated familiarity level.
Intercultural analogy	Reduce cultural distance without oversimplification	Offer one brief analogy to a familiar learning context while explicitly noting that the analogy is partial and context-specific.
Reflection prompt	Encourage transfer-oriented interpretation	Ask the learner to explain the symbol to a person from another cultural background, focusing on meaning, context, and respectful interpretation.
Quality guardrail	Preserve accuracy and coherence	Keep each response concise, avoid stereotypes, distinguish widely recognized interpretations from local variations, and maintain narrative continuity.

Table A4. Measurement items used for the focal constructs.

Construct	Items
Behavioral engagement	I stayed focused during the learning activity; I made a serious effort to follow the explanations; I persisted when the cultural meaning was not immediately clear.
Emotional engagement	I felt interested in the cultural symbols; the learning activity made the symbols feel vivid and meaningful; I felt personally involved in the story-based learning process.
Cognitive engagement	I actively tried to interpret deeper meanings; I connected the symbols with historical or social contexts; I reflected on how the symbols could be explained to people from another background.
Perceived GenAI-based storytelling quality	The AI-supported story adjusted explanations to my understanding needs; the story made the cultural symbols vivid; the explanations were context-sensitive; the AI-supported story helped me interpret cultural meanings.
Cross-cultural learning outcomes	I can explain the symbolic meaning of the cultural materials; I understand the cultural context behind the symbols; I feel confident interpreting similar symbols; I can remember the key meanings; I can apply what I learned to new intercultural situations; I can explain these symbols respectfully to someone from another background.

emphasize symbolic explanation, contextual understanding, retention, and transfer, thereby matching the paper's definition of cross-cultural learning.

Application to Chinese cultural-symbol teaching. These items can be adapted for Chinese cultural learning evaluation by replacing the general phrase "cultural symbols" with the specific content of a lesson, such as dragon motifs, opera masks, Chinese knots, festival colors, calligraphy forms, or architectural patterns. Teachers can use the engagement items as a short classroom feedback tool and the learning-outcome items as a post-activity reflection scale to assess whether students can explain Chinese cultural symbols to audiences from different cultural backgrounds.

Appendix D. Transfer-Scenario Scoring Rubric

Table A5. Transfer-scenario scoring rubric.

Score	Descriptor
0	No interpretable response or a response unrelated to the cultural symbol.
1	Surface recognition only; the response names a visible feature but does not explain cultural meaning.
2	Basic meaning is identified, but the explanation is vague, decontextualized, or partly inaccurate.
3	Culturally relevant meaning is explained with acceptable contextual accuracy but limited interpretive depth.
4	Meaning, context, and social or historical significance are explained clearly, with appropriate attention to the intended audience.
5	The response provides accurate, contextualized, and transferable interpretation while avoiding stereotyping and acknowledging cultural specificity.

Interpretive analysis. Table A5 operationalizes transfer performance as more than factual recall. Higher scores require learners to connect visible features with social, historical, and audience-sensitive interpretation. This rubric therefore matches the study's argument that cross-cultural symbol learning should be evaluated by learners' ability to explain meaning in a new intercultural context, not only by their ability to recognize an object or repeat a definition.

Application to Chinese cultural-symbol teaching. In Chinese cultural education, the rubric can be used for short written responses, oral presentations, museum-guide role-play activities, or intercultural dialogue tasks. For example, learners can be asked to explain the meaning of a Peking opera mask or a Mid-Autumn moon image to a peer who is unfamiliar with Chinese cultural conventions. The rubric helps instructors distinguish surface recognition from culturally responsible interpretation, which is central to teaching Chinese cultural symbols in international classrooms.

Appendix E. GenAI Output Quality Screening

Interpretive analysis. Table A6 specifies the common criteria used during both pre-deployment validation and post-deployment screening. The criteria address educational quality and cultural responsibility: a response needed to preserve approved meanings, remain coherent, avoid uncontrolled expansion, and support interpretation rather than generic content production. Applying the same decision framework before deployment and to a random sample of study logs strengthens the internal validity of the GenAI condition by showing how adaptive interaction was kept within a controlled instructional boundary.

Table A6. Quality-screening criteria for GenAI-generated responses.

Criterion	Screening decision rule
Cultural accuracy	The response preserves the approved symbolic meaning and does not introduce unsupported historical claims.
Narrative coherence	The response remains connected to the learning scene and does not fragment the story into unrelated facts.
Boundary control	The response stays within the approved instructional core and does not expand exposure time or content scope substantially beyond the comparison conditions.
Intercultural responsibility	The response avoids cultural stereotypes, false equivalence, and essentialist claims about any group.
Learning alignment	The response supports interpretation, clarification, or transfer rather than simply producing decorative or generic content.

Application to Chinese cultural-symbol teaching. These screening criteria can be adopted by instructors who use GenAI to teach Chinese culture. Before using AI-generated cultural explanations in class, teachers can check whether the output preserves recognized cultural meanings, avoids stereotypes or false equivalences, and supports learners' ability to interpret and explain cultural context. In classroom practice, this screening process is especially important when teaching symbolically dense Chinese materials, because overgeneralized or inaccurate AI explanations may weaken learners' intercultural understanding.
